# circ_0000376 knockdown suppresses non-small cell lung cancer cell tumor properties by the miR-545-3p/PDPK1 pathway

**DOI:** 10.1515/med-2023-0641

**Published:** 2023-02-16

**Authors:** Changpeng Sun, Hongjun Guan, Jinjin Li, Yinfeng Gu

**Affiliations:** Department of Cardiothoracic Surgery, Jianhu Clinical Medical College of Yangzhou University, No. 666, Nanhuan Road, Jinhu Town, Jianhu, Yancheng City, Jiangsu Province, 224700, PR China; Department of Cardiothoracic Surgery, Jianhu Clinical Medical College of Yangzhou University, Yancheng City, Jiangsu Province, 224700, PR China

**Keywords:** NSCLC, circ_0000376, miR-545-3p, PDPK1

## Abstract

Non-small cell lung cancer (NSCLC) accounts for 80% of total lung cancers, which are the main killer of cancer-related death worldwide. Circular RNA (circRNA) has been found to modulate NSCLC development. However, the role of circ_0000376 in NSCLC development has been underreported. The present work showed that circ_0000376 and 3-phos-phoinositide-dependent protein kinase-1 (PDPK1) expression were dramatically increased, but miR-545-3p was decreased in NSCLC tissues and cells. circ_0000376 expression was closely associated with lymph node metastasis, tumor-node-metastasis stage, and tumor size of NSCLC patients. circ_0000376 knockdown repressed NSCLC cell proliferation, migration, invasion, and glutaminolysis but induced cell apoptosis. Additionally, miR-545-3p bound to circ_0000376, and circ_0000376 regulated cell phenotypes by associating with miR-545-3p. MiR-545-3p also participated in NSCLC cell proliferation, migration, invasion, apoptosis, and glutaminolysis by targeting PDPK1. Further, circ_0000376 absence repressed tumor formation *in vivo*. Collectively, circ_0000376 regulated NSCLC cell tumor properties by the miR-545-3p/PDPK1 axis, suggesting that circ_0000376 could be employed as a therapeutic target for NSCLC.

## 1 Introduction

Lung cancer ranks first in mortality in men and accounts for over 10% of total cancers globally [1,2]. More than 80% of lung carcinomas are identified as non-small cell lung cancer (NSCLC) [3]. Despite much progress in NSCLC treatment, the prognosis of NSCLC cases is poor [4]. The dysregulation of circular RNA (circRNA) has been considered as the leading cause of NSCLC progression [5], but the precise inner mechanism is still underreported. Therefore, a deep understanding of the pathogenesis of NSCLC will be pressing to seek reliable and effective therapeutic targets for the cancer.

CircRNA is a specific transcript that forms a continuous loop structure, featured by conservatism, stability, and specificity [6]. CircRNA commonly functions by serving as a sponge of microRNA (miRNA). Most notably, circRNA CDR1 contains over 70 complementary sites of miR-7 [7]. Previous data have indicated the potential of circRNA as a new clinical biomarker and potential target in various cancers [8]. CircRNA is also involved in various cancer development, such as gastric carcinoma [9], bladder carcinoma [10], ovarian cancer [11], and NSCLC [12]. circ_0000376 has been revealed to facilitate gastric cancer tumorigenesis [13]. Besides, Peng and his colleagues explained that circ_0000376 might be involved in breast cancer development by regulating miR-1285-3p [14]. circ_0000376 is one of the 16 upregulated circRNAs between NSCLC tissues and adjacent normal tissues [15]. But the role of circ_0000376 in NSCLC progression has not been fully revealed.

MiRNA is a highly conserved non-coding RNA that possesses nearly 20 nucleotides and controls gene expression through binding to its non-coding region [16]. An increasing number of studies have underlined the core role of miRNA in governing NSCLC progression [17,18]. For example, miRNA can simultaneously activate the Wnt/β-catenin pathway to regulate NSCLC cell metastasis [19]. MiRNA also regulates the sensitivity of NSCLC to chemotherapy [20] and radiotherapy [21]. As reported, miR-545-3p acted as a suppressor in the NSCLC process [22]. 3-Phos-phoinositide-dependent protein kinase-1 (PDPK1) belongs to the AGC serine/threonine kinase family [23]. PDPK1 can activate many downstream effectors and thereby facilitates cancer progression [24]. Moreover, PDPK1 participated in NSCLC cell proliferation and invasion [25].

As predicted, miR-545-3p concurrently contained the complementary sites of circ_0000376 and PDPK1. Thus, we hypothesized that circ_0000376 induced PDPK1 by binding to miR-545-3p to mediate NSCLC progression. However, the published data have not reported whether the circ_0000376/miR-545-3p/PDPK1 was involved in the development of NSCLC. The present work analyzed the effects of circ_0000376 on NSCLC cell tumor properties and explored whether circ_0000376 participated in NSCLC development through the miR-545-3p-dependent PDPK1.

## 2 Materials and methods

### 2.1 Specimen collection

NSCLC tissues (*N* = 30) and adjacent healthy lung tissues (*N* = 30) were collected from NSCLC sufferers at Jianhu Clinical Medical College of Yangzhou University. The obtained tissues were kept at −80°C. The subjects signed the written informed consent before operation. The Ethics Committee of Jianhu Clinical Medical College of Yangzhou University approved this study.

### 2.2 Cell purchase and culture

Procell (Wuhan, China) provided human bronchial epithelial cell line (16HBE), lung adenocarcinoma cell line (H522), and NSCLC cell line (A549). 16HBE and H522 cells were grown in RPMI-1640 (Procell), while A549 cells were cultured in Ham’s F12K (Procell) at 37°C in a humid incubator with 5% CO_2_. RPMI-1640 and Ham’s F12K were supplemented with 10% fetal bovine serum (FBS; Procell) and 1% penicillin/streptomycin (Procell).

### 2.3 Oligonucleotides synthesis and plasmid construction

Small interfering RNA against circ_0000376 (si-circ_0000376, 5′-TCCATATGAGAGTTGGATTCT-3′; si-circ_0000376#2 5′-TATCCATATGAGAGTTGGATT-3′), the small hairpin RNA against circ_0000376 (sh-circ_0000376), miR-545-3p mimic (5′-UCAGCAAACAUUUAUUGUGUGC-3′), miR-545-3p inhibitor (5′-GCACACAAUAAAUGUUUGCUGA-3′), the overexpression plasmid of PDPK1 (pc-PDPK1), and controls (si-NC, sh-NC, miR-NC, miRNA inhibitor-NC, and pc-NC) were provided by Ribobio Co., Ltd (Guangzhou, China). Cell transfection was performed using TurboFect reagent (Thermo Fisher, Waltham, MA, USA) based on the instruction of the manufacturer.

### 2.4 Quantitative real-time PCR (qRT-PCR) and RNA treatment

RNAsimple reagents (Tiangen, Beijing, China) were used for RNA extraction. RNA was incubated with RNase R (Xiyuan Biotechnology, Shanghai, China) to analyze circ_0000376 stability, regarding untreated cells as controls (mock). Reverse transcription was carried out using FastKing RT Kit (Tiangen) and MicroRNA RT reagents (Thermo Fisher). Then, FastFire qPCR PreMix (Tiangen) was utilized to analyze gene expression. Finally, results were analyzed with the 2^−∆∆Ct^ method with GAPDH and U6 as controls. The primer sequences are displayed as follows: circ_0000376 5′-ATGAAGGCTAGTTTGGAT-3′ and 5′-TAGTCAGGCATAGTGAAG-3′; miR-545-3p 5′-ACACTCCAGCTGGGTCAGCAAACATTTATT-3′ and 5′-TGGTGTCGTGGAGTCG-3′; PDPK1 5′-AGCATCAGTCCGAACCAT-3′ and 5′-GAGTTCCAGGACCACAGC-3′.

### 2.5 Cell counting kit-8 (CCK-8)

In brief, H522 and A549 cells were cultured in 96-well plates (8 × 10^3^ cells per well) and transfected with siRNAs of circ_0000376, si-NC, miR-545-3p inhibitor, miRNA inhibitor-NC, miR-545-3p mimic, miR-NC, pc-PDPK1, and pc-NC. After 48 h, CCK-8 solution (Abcam, Cambridge, UK) was added to each well. After 3 h, these samples were analyzed with a microplate reader (Thermo Fisher).

### 2.6 Cell colony formation assay

H522 and A549 cells were seeded in six-well plates and transfected with plasmids and oligonucleotides. After about 2-week culture, the cells were fixed with paraformaldehyde and dyed with crystal violet. Finally, cell colony-forming ability was determined by counting the number of positive colonies.

### 2.7 Transwell migration and invasion assays

NSCLC cells were mixed with serum-free RPMI-1640 (Procell) or Ham’s F12K (Procell) and then added into the upper chambers, which were coated with Matrigel (Qcbio Science, Shanghai, China) for cell invasion assay. Accordingly, RPMI-1640 containing 15% FBS (Procell) as well as Ham’s F12K possessing 15% FBS were placed into the lower chambers. After 24 h, cells were stained using crystal violet. Finally, cell migratory and invasive capacities were determined under a microscope (Olympus, Tokyo, Japan) with a 100(×) magnification.

### 2.8 Wound-healing assay

H522 and A549 cells were transfected with plasmids and oligonucleotides. About 2-week culture, cell wounds were created, and FBS-free RPMI-1640 (Procell) and Ham’s F12K (Procell) were added to the culture wells. After 24 h, results were analyzed under a microscope (Olympus).

### 2.9 Flow cytometry analysis

Cells were harvested after digestion with trypsin (Thermo Fisher) and homogenized in binding buffer (Solarbio, Beijing, China). Afterward, the cells were incubated with Annexin V-FITC (Solarbio) and propidium iodide (PI) (Solarbio), in the dark. These samples were assessed with flow cytometry (Thermo Fisher).

### 2.10 Glutamate detection assay

H522 and A549 cells were grown in 4.5 cm petri dishes and transfected with plasmids and oligonucleotides. After 48 h, cell samples were lysed with Mammalian Lysis Buffer (Abcam) and then incubated with Enzyme Mixture (Abcam) and nicotinamide adenine dinucleotide phosphate stock solution (Abcam). Finally, these samples were assessed using an enzyme immunoassay analyzer (Thermo Fisher).

### 2.11 Glutamine determination assay

Briefly, cells were harvested and homogenized in cold Hydrolysis Buffer (Abcam). Insoluble material was removed by centrifugation, and the supernatant was transferred into a clean tube. After that, the samples were purified with perchloric acid (Sigma, St. Louis, MO, USA) and potassium hydroxide, and finally analyzed with an enzyme immunoassay analyzer (Thermo Fisher).

### 2.12 Western blot analysis

Lysates obtained using NP-40 lysis buffer were loaded onto SDS-PAGE gels (Phygene, Fuzhou, China), and protein bands were electrotransferred onto nitrocellulose membranes. After that, the membranes were blocked with defatted dry milk (Solarbio) and incubated with anti-proliferating cell nuclear antigen (anti-PCNA; 1:1,000), anti-BCL2-associated x protein (anti-Bax; 1:5,000), anti-glutaminase (GLS1; 1:5,000), anti-PDPK1 (1:1,000) as well as anti-GAPDH (1:20,000). The membranes were incubated with secondary antibodies (1:5,000), and protein bands were developed with RapidStep ECL Reagent (Millipore, Bradford, MA, USA). All antibodies were obtained from Abcam Co., Ltd. The intensity of protein bands was quantified by image J software. The relative protein expression was normalized to GAPDH.

### 2.13 Dual-luciferase reporter assay

The putative binding sites between miR-545-3p and circ_0000376 or PDPK1 were predicted by circular RNA interactome (https://circinteractome.nia.nih.gov/api/v2/mirnasearch? circular_rna_query = hsa_circ_0000376&mirna_query = hsa-miR-545&submit = miRNA + Target + Search) or targetscan online database (http://www.targetscan.org/cgi-bin/targetscan/vert_72/view_gene.cgi? rs = ENST00000441549.3&taxid = 9606&members = miR-545-3p&showcnc = 1&shownc = 1&showncf1 = 1&showncf2 = &subset = 1). Based on the complementary sites, the wild-type (WT) plasmids of circ_0000376 (circ_0000376 WT) and the 3′-untranslated region (3′-UTR) of PDPK1 (PDPK1-3′-UTR WT) and mutant (MUT) plasmids (circ_0000376 MUT and PDPK1-3′-UTR MUT) were built by Geneseed (Guangzhou, China). Subsequently, the above plasmids were mixed with miR-545-3p mimic or miR-NC and transfected into H522 and A549 cells. After 48 h, luciferase activity was analyzed with the Dual-Lucy Assay Kit (Solarbio).

### 2.14 *In vivo* assay

The assay was carried out on 12 male BALB/c nude mice (6 weeks, weighting 20 ± 2 g) purchased from Laboratory Animal, Inc. (Beijing, China). 5 × 10^6^ A549 cells were hypodermically injected into the center of the back of mice. After 7 days, tumor volume was measured. After 28 days, the mice were euthanized by cervical dislocation, and the tumor tissues were harvested for further analysis. The Animal Care and Use Committee of Jianhu Clinical Medical College of Yangzhou University approved this study.

### 2.15 Immunohistochemistry (IHC) assay

IHC assay was conducted to analyze the protein expression of nuclear proliferation marker (Ki67) and PDPK1 in xenografts. In brief, the tissues were cut into sections, fixed with paraformaldehyde (Sigma), dehydrated with ethanol (Millipore), and embedded into paraffin. Next, the sections were dewaxed using xylene (Millipore) and hydrated with ethanol. Then, the primary antibody against Ki67 (1:200; Abcam) or PDPK1 (1:150; Abcam) was employed to incubate the tissues. After being washed by PBS (Solarbio), these sections were reacted with secondary antibodies. Finally, staining images were photographed under a microscope. Meanwhile, for negative controls, the primary antibodies were replaced by PBS under the same conditions.

### 2.16 Statistical analysis

Data were collected based on three independent duplicate tests and analyzed with SPSS software. Results were shown as mean ± standard deviation. Significant differences were compared with Student’s *t*-tests, Wilcoxon rank-sum test, or analysis of variance. Fisher’s exact test was used for comparing groups between low and high circ_0000376 expression. *P*-value <0.05 indicated a significant difference.

## 3 Results

### 3.1 circ_0000376 expression was upregulated in NSCLC tissues and cells

**Figure 1:**
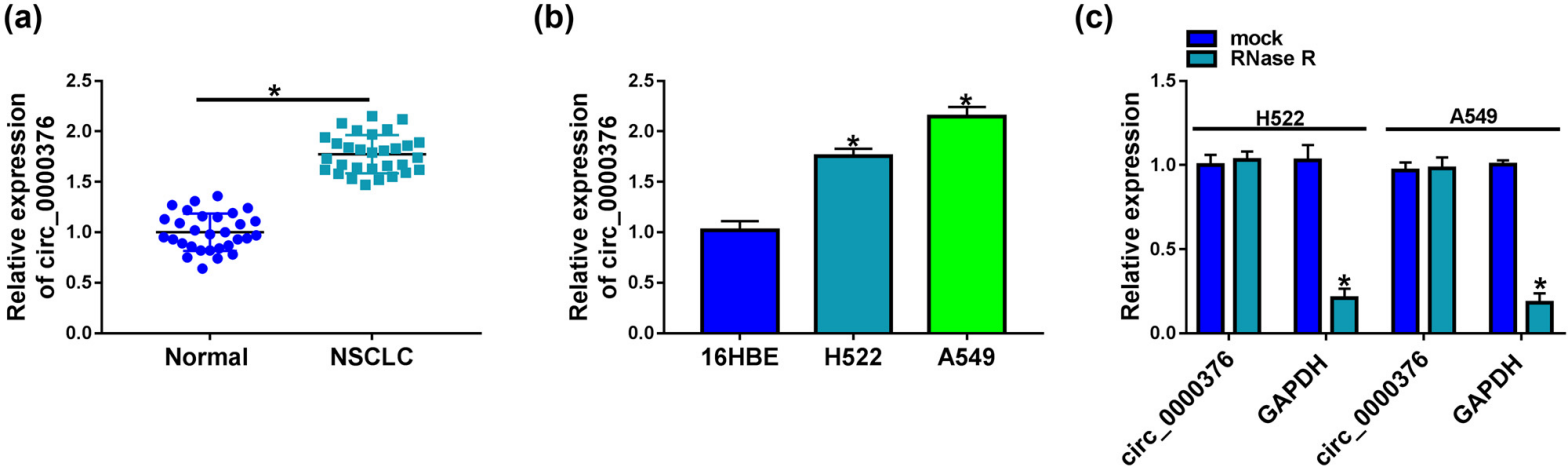
circ_0000376 was upregulated in NSCLC tissues and cells. (a and b) circ_0000376 expression was detected by qRT-PCR in 30 pairs of NSCLC tissues and paracancerous normal lung tissues as well as 16HBE, H522, and A549 cells. (c) RNase R resistance analysis assay showed that circ_0000376 was more stable than linear GAPDH. *P < 0.05.

circ_0000376 expression was first detected in NSCLC tissues, and the results showed that circ_0000376 was overexpressed in NSCLC tissues compared with paracancerous normal lung tissues (Figure 1a). circ_0000376 expression was significantly increased in H522 and A549 cells when compared with 16HBE cells (Figure 1b). As presented in Table 1, circ_0000376 expression was closely associated with lymph node metastasis, tumor-node-metastasis (TNM) stage, and tumor size of NSCLC patients. Additionally, we found that circ_0000376 expression had no significant difference after treatment of RNase R, but GAPDH expression was dramatically downregulated (Figure 1c), suggesting that circ_0000376 was more stable than linear GAPDH. These data demonstrated that circ_0000376 might be involved in the pathogenesis of NSCLC.

**Table 1:** Association between circ_0000376 expression and clinical features of NSCLC patients

Clinical feature	circ_0000376 expression		*P*
	High (*N* = 15)	Low (*N* = 15)	
Age			>0.05
≤50 years	7	8	
>50 years	8	7	
Gender			
Female	10	6	>0.05
Male	5	9	
Lymph node metastasis			<0.05
Yes	12	5	
No	3	10	
TNM stage			<0.05
I + II	2	11	
III + IV	13	4	
Tumor size (cm)			<0.05
≤3	5	11	
>3	10	4	

### 3.2 circ_0000376 silencing repressed cell proliferation, migration, invasion, and glutamine catabolism and induced cell apoptosis in H522 and A549 cells

**Figure 2:**
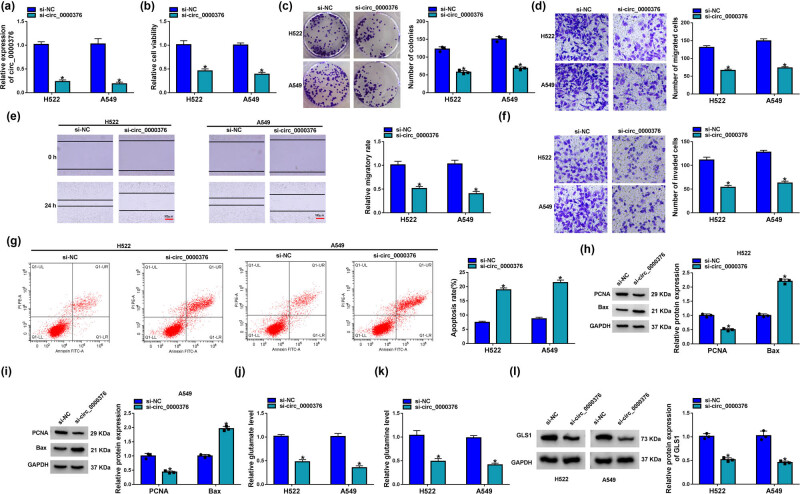
circ_0000376 absence repressed NSCLC cell tumor properties. (a–l) Both H522 and A549 cells were transfected with si-NC and si-circ_0000376, respectively. (a) Interfering efficiency of si-circ_0000376 was determined by qRT-PCR. (b and c) Cell viability and colony-forming ability were detected by CCK-8 and colony formation assays, respectively. (d and e) Transwell migration and wound-healing assays were performed to analyze the migratory ability of H522 and A549 cells. (f) Transwell invasion assay was carried out to determine the invasive ability of H522 and A549 cells. (g) Cell apoptosis was detected by flow cytometry analysis. (h, i, and l) PCNA, Bax, and GLS1 protein expression were detected by western blot analysis. (j and k) Levels of glutamate and glutamine were determined by glutamate detection and glutamine determination assays, respectively. *P < 0.05.

The study then analyzed whether circ_0000376 participated in NSCLC process *in vitro*. Based on the high expression of circ_0000376 in H522 and A549 cells, the small interfering RNA against circ_0000376 was synthesized and its efficiency in downregulating circ_0000376 expression was then determined. Data from qRT-PCR analysis displayed that circ_0000376 expression was dramatically downregulated after transfection of si-circ_0000376 and si-circ_0000376#2 (Figure 2a and Figure S1a). Subsequently, circ_0000376 silencing inhibited H522 and A549 cell viability and colony-forming ability (Figure 2b and c and Figure S1b and c), which suggested that the reduced expression of circ_0000376 could hinder cell proliferation. The migration and invasion of H522 and A549 cells were also inhibited after the downregulation of circ_0000376 (Figure 2d–f and Figure S1d–f). Additionally, the apoptosis of H522 and A549 cells was promoted by the decreased expression of circ_0000376 (Figure 2g and Figure S1g). PCNA is an essential protein in DNA replication and repair and functions by anchoring DNA polymerases and DNA editing enzymes, inhibiting tumor cell proliferation [26]. Bax is a pro-apoptotic protein that possesses 9 α-helices to the mitochondria during cell apoptosis, inhibiting tumor development [27]. circ_0000376 depletion downregulated PCNA protein expression and upregulated Bax protein expression (Figure 2h and i). Further, circ_0000376 knockdown suppressed the production of glutamate and glutamine (Figure 2j and k and Figure S1h and i). In order to further determine the effect of circ_0000376 downregulation on glutamine metabolism, GLS1 protein expression was analyzed in the H522 and A549 cells transfected with si-circ_0000376 and si-NC. Western blot analysis revealed that circ_0000376 absence decreased GLS1 protein level (Figure 2l). Collectively, the above evidence demonstrated that circ_0000376 knockdown inhibited NSCLC cell proliferation, migration, invasion, and glutamine metabolism and induced cell apoptosis.

### 3.3 circ_0000376 was associated with miR-545-3p in H522 and A549 cells

**Figure 3:**
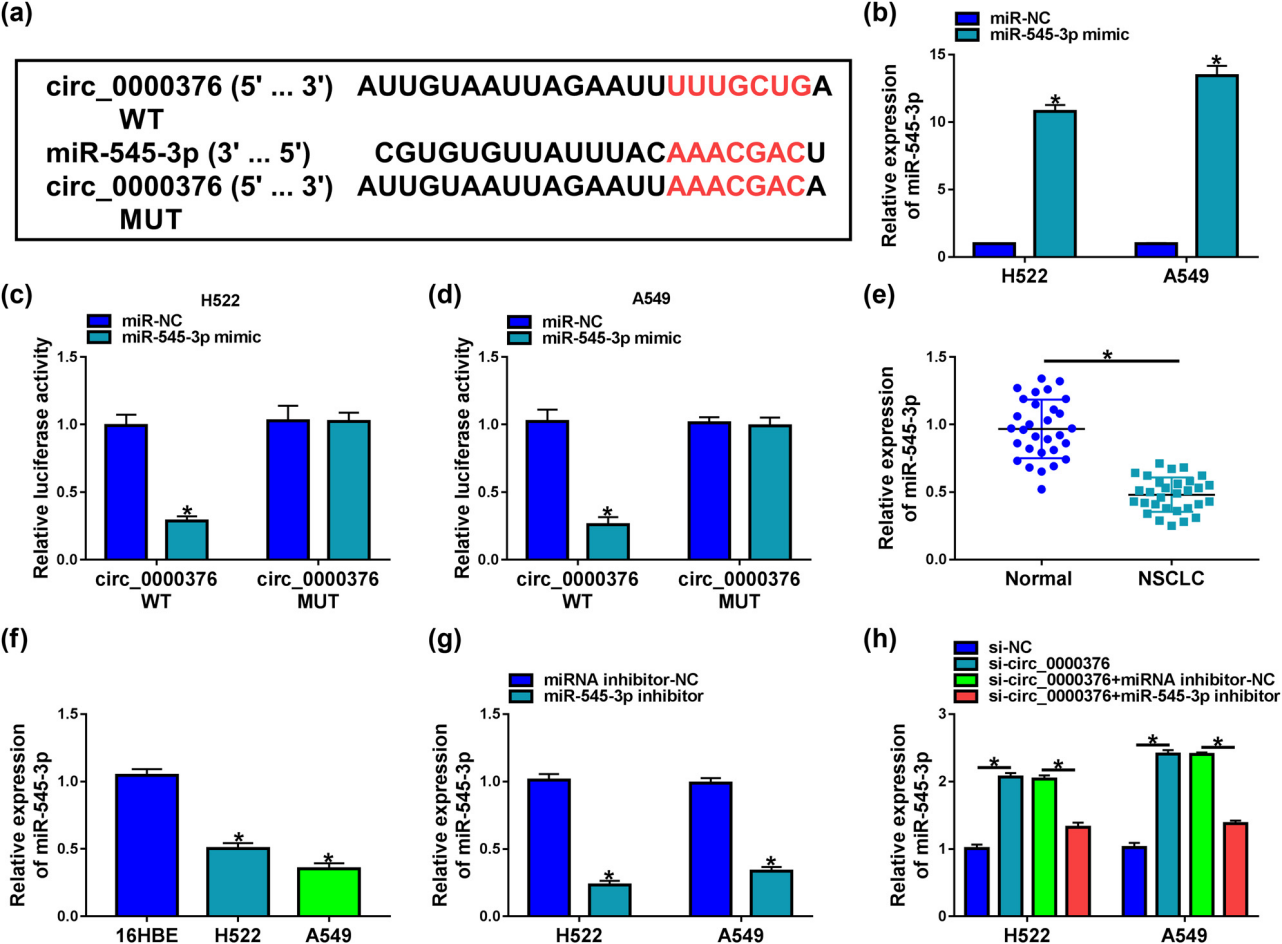
circ_0000376 interacted with miR-545-3p in H522 and A549 cells. (a) Binding sites between circ_0000376 and miR-545-3p were predicted by the circular RNA interactome online database. (b and g) Efficiency of miR-545-3p mimic and inhibitor in increasing or decreasing miR-545-3p was determined by qRT-PCR in H522 and A549 cells. (c and d) Dual-luciferase reporter assay was performed to demonstrate that circ_0000376 was associated with miR-545-3p in H522 and A549 cells. (e and f) MiR-545-3p expression was determined by qRT-PCR in 30 pairs of NSCLC tissues and paracancerous normal lung tissues as well as 16HBE, H522, and A549 cells. (h) Impacts between circ_0000376 knockdown and miR-545-3p inhibitor on miR-545-3p expression were disclosed by qRT-PCR in H522 and A549 cells. *P < 0.05.

To disclose the mechanism by which circ_0000376 mediated NSCLC cell tumor properties, the miRNA interacted with circ_0000376 was further searched. As predicted by the circular RNA interactome online database, the circ_0000376 sequence contained the complementary sites of the miR-545-3p sequence (Figure 3a), implying that circ_0000376 might be associated with miR-545-3p. The result exhibited that miR-545-3p mimic was effective in increasing miR-545-3p expression (Figure 3b). As shown in Figure 3c and d, miR-545-3p mimic dramatically repressed the relative luciferase activity of circ_0000376 WT but not that of circ_0000376 MUT. Additionally, we found that miR-545-3p expression was downregulated in NSCLC tissues and H522 and A549 cells in comparison with the matched normal lung tissues and 16HBE cells, respectively (Figure 3e and f). Further, the effects between circ_0000376 silencing and miR-545-3p inhibitor on miR-545-3p expression were determined. As presented in Figure 3g, miR-545-3p inhibitor was effective in decreasing miR-545-3p expression. It was then found that miR-545-3p expression was upregulated after knockdown of circ_0000376, whereas this effect was attenuated by miR-545-3p inhibitor (Figure 3h). All in all, these data demonstrated that circ_0000376 was associated with miR-545-3p in H522 and A549 cells.

### 3.4 circ_0000376 regulated H522 and A549 cell proliferation, migration, invasion, apoptosis, and glutamine catabolism by binding to miR-545-3p

**Figure 4:**
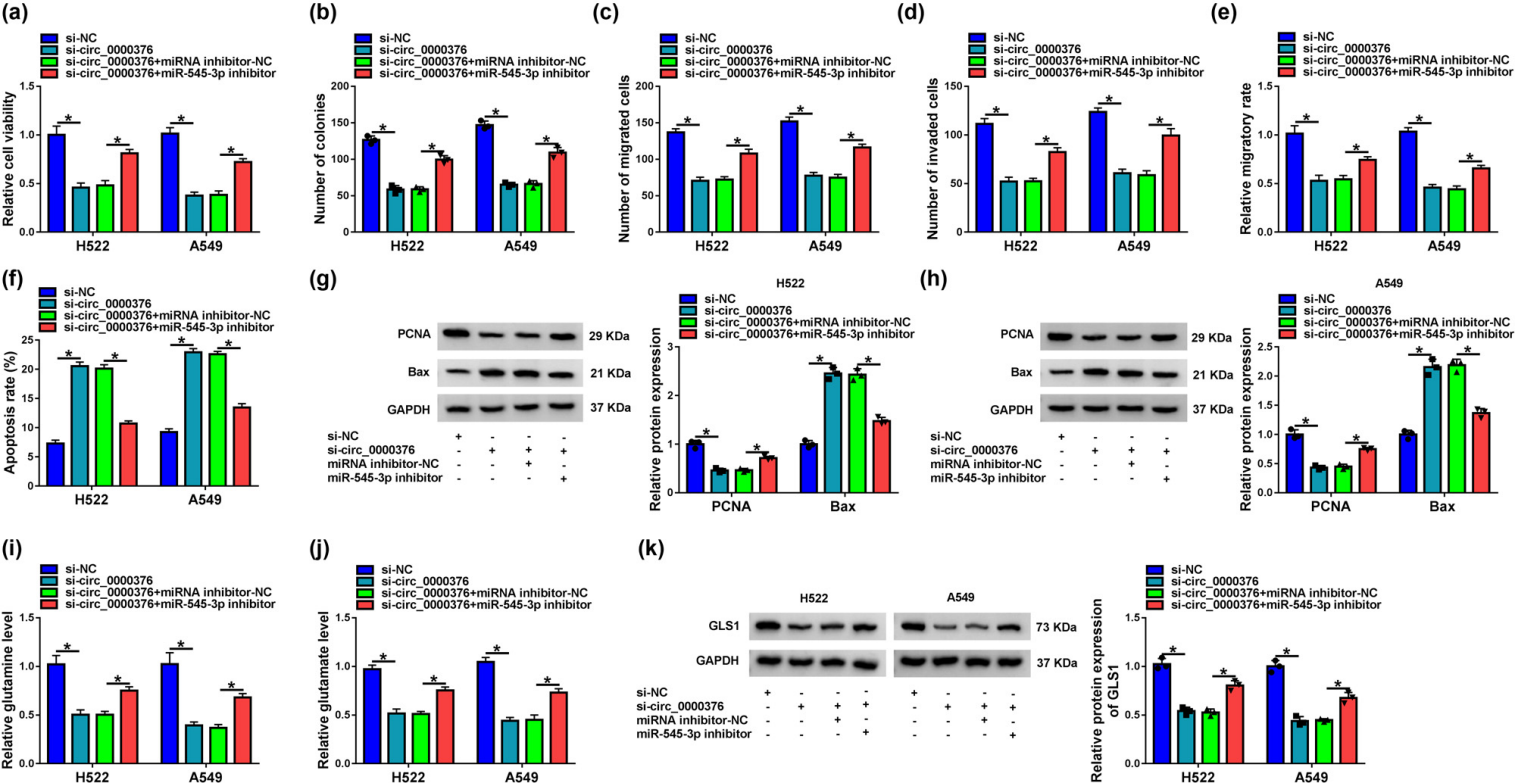
circ_0000376 knockdown repressed NSCLC cell tumor properties by binding to miR-545-3p. (a–k) Both H522 and A549 cells were transfected with si-NC, si-circ_0000376, si-circ_0000376 + miRNA inhibitor-NC, and si-circ_0000376 + miR-545-3p inhibitor, respectively. (a and b) Cell viability and colony-forming ability were detected by CCK-8 assay and colony formation assay, respectively. (c and e) Transwell migration and wound-healing assays were performed to assess the migratory ability of H522 and A549 cells. (d) Transwell invasion assay was conducted to determine the invasive ability of H522 and A549 cells. (f) Cell apoptosis was detected by flow cytometry analysis. (g and h) Western blot analysis was performed to detect the protein expression of PCNA and Bax. (i and j) Levels of glutamine and glutamate were determined by glutamine determination and glutamate detection assays, respectively. (k) GLS1 protein expression was detected by western blot analysis. *P < 0.05.

Whether circ_0000376 regulated NSCLC cell tumor properties by binding to miR-545-3p was explored in this part. The results showed that circ_0000376 silencing repressed H522 and A549 cell viability and colony-forming ability, whereas these effects were restored by miR-545-3p inhibitor (Figure 4a and b). The repressive impacts of circ_0000376 silencing on the migration and invasion of H522 and A549 cells were also restrained by miR-545-3p inhibitor (Figure 4c–e). Additionally, the promoting effect of circ_0000376 knockdown on cell apoptosis was reversed after transfection of miR-545-3p inhibitor (Figure 4f). In line with the above data, the impacts of circ_0000376 silencing on the protein expression of PCNA and Bax were partially reversed after the downregulation of miR-545-3p (Figure 4g and h). circ_0000376 knockdown-mediated repressive impacts on glutamine and glutamate production as well as GLS1 protein expression were partly abolished by miR-545-3p inhibitor (Figure 4i–k). Thus, these results demonstrated that circ_0000376 could modulate H522 and A549 cell phenotypes by binding to miR-545-3p.

### 3.5 circ_0000376 regulated PDPK1 by interacting with miR-545-3p in H522 and A549 cells

**Figure 5:**
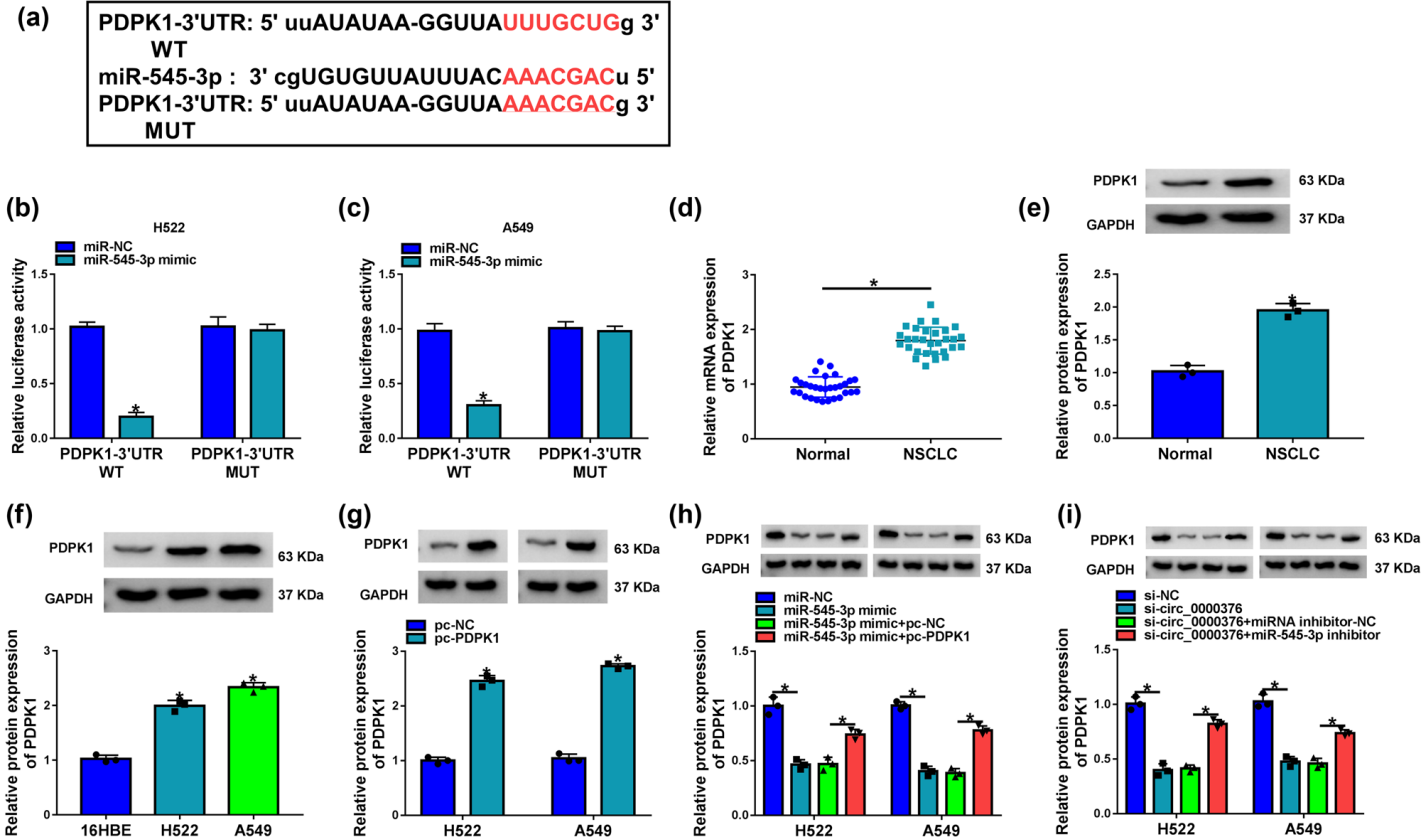
circ_0000376 modulated PDPK1 expression by associating with miR-545-3p. (a) Putative binding sites of miR-545-3p for PDPK1 were predicted by the targetscan online database. (b and c) Dual-luciferase reporter assay was performed to demonstrate that miR-545-3p was associated with PDPK1 in H522 and A549 cells. (d) PDPK1 mRNA expression was determined by qRT-PCR in 30 pairs of NSCLC tissues and paracancerous normal lung tissues. (e and f) PDPK1 protein expression was detected by western blot analysis in 30 pairs of NSCLC tissues and paracancerous normal lung tissues as well as 16HBE, H522, and A549 cells. (g) PDPK1 protein expression was determined by western blot analysis in H522 and A549 cells transfected with pc-PDPK1 or pc-NC. (h) Impacts between miR-545-3p mimic and PDPK1 overexpression on PDPK1 protein expression were revealed by western blot analysis in H522 and A549 cells. (i) Effects between circ_0000376 knockdown and miR-545-3p inhibitor on PDPK1 protein expression were determined by western blot analysis in H522 and A549 cells. *P < 0.05.

The targetscan online database showed that PDPK1 contained the binding sequence of miR-545-3p (Figure 5a). Dual-luciferase reporter assay showed that miR-545-3p mimic dramatically repressed the relative luciferase activity of PDPK1-3′-UTR WT, whereas it did not repress that of PDPK1-3′-UTR MUT (Figure 5b and c). Subsequently, PDPK1 mRNA expression was substantially upregulated in NSCLC tissues when compared with paracancerous normal lung tissues (Figure 5d). The protein expression of PDPK1 was also increased in NSCLC tissues as well as H522 and A549 cells in comparison with adjacent normal lung tissues and 16HBE cells, respectively (Figure 5e and f). Additionally, PDPK1 expression was significantly increased in H522 and A549 cells transfected with the overexpression plasmid of PDPK1 (Figure 5g), suggesting that pc-PDPK1 was effective in increasing PDPK1 expression. PDPK1 protein expression was downregulated by miR-545-3p mimic, but this impact was attenuated after PDPK1 overexpression (Figure 5h). These results demonstrated that miR-545-3p targeted PDPK1 in H522 and A549 cells. Further, circ_0000376 silencing notably reduced the PDPK1 level, while miR-545-3p inhibitor reversed this impact (Figure 5i), suggesting that circ_0000376 could control PDPK1 expression by binding to miR-545-3p.

### 3.6 MiR-545-3p mimic repressed NSCLC cell tumor properties by targeting PDPK1

**Figure 6:**
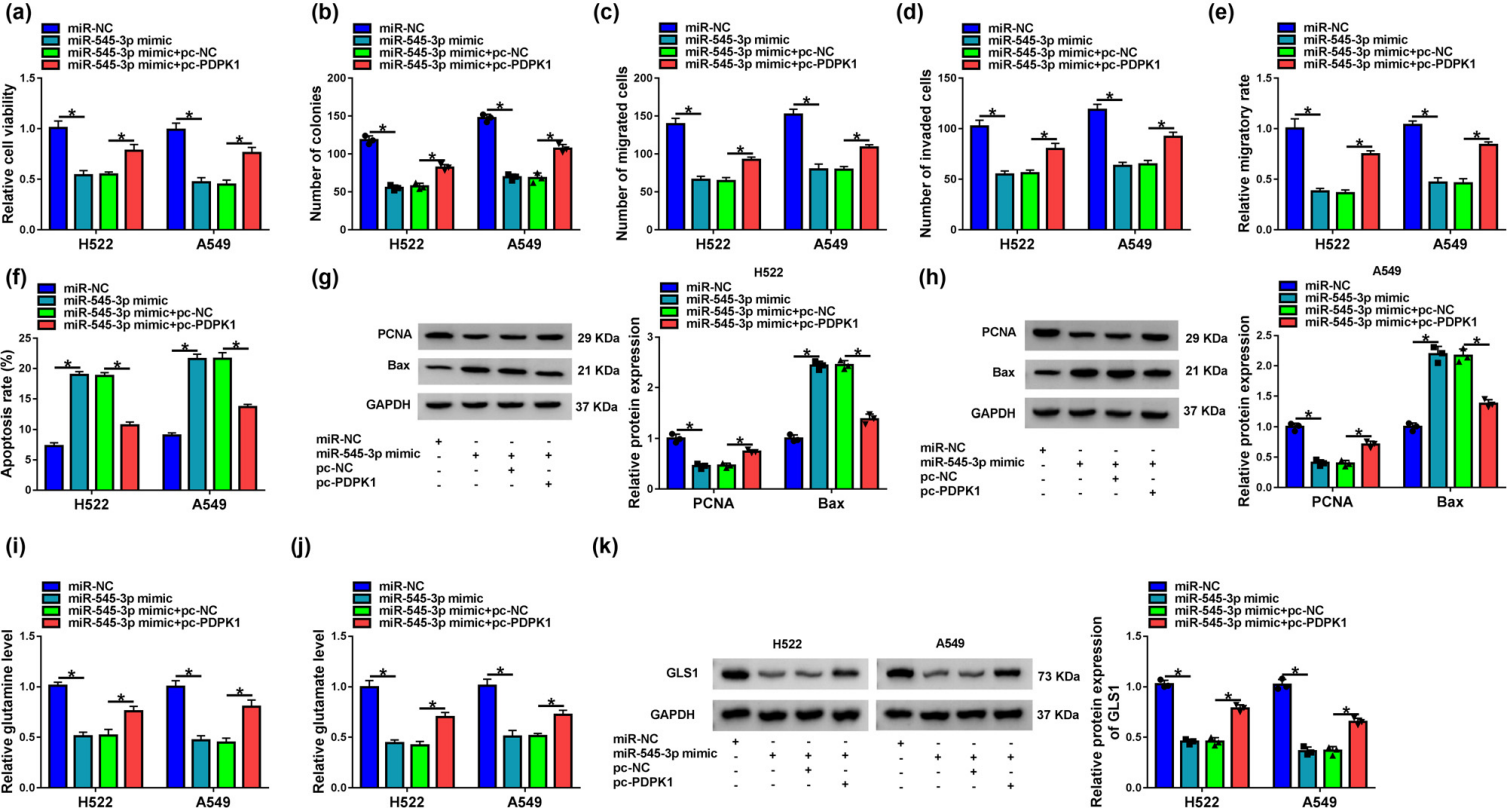
MiR-545-3p mimic inhibited H522 and A549 cell tumor properties by associating with PDPK1. (a–k) MiR-NC, miR-545-3p mimic, miR-545-3p mimic + pc-NC, and miR-545-3p mimic + pc-PDPK1 were transfected into H522 and A549 cells. (a and b) CCK-8 and colony formation assays were employed to detect cell viability and colony-forming ability, respectively. (c and e) Transwell migration and wound-healing assays were carried out to evaluate the migratory ability of H522 and A549 cells. (d) Transwell invasion assay was conducted to determine the invasive ability of H522 and A549 cells. (f) Cell apoptosis was determined by flow cytometry analysis. (g and h) Protein expression of PCNA and Bax was determined by western blot analysis. (i and j) Levels of glutamine and glutamate were determined by glutamine determination and glutamate detection assays, respectively. (k) GLS1 protein expression was detected by western blot analysis. *P < 0.05.

Given the association between miR-545-3p and PDPK1, we further determined whether PDPK1 participated in miR-545-3p-mediated actions in H522 and A549 cells. Results showed that miR-545-3p suppressed cell viability and colony-forming ability, whereas these effects were reversed after PDPK1 overexpression (Figure 6a and b). The migration and invasion of H522 and A549 cells were also inhibited by miR-545-3p mimic, which was attenuated by PDPK1 overexpression (Figure 6c–e). Additionally, the promoting effect of miR-545-3p mimic on cell apoptosis was restrained by ectopic PDPK1 expression (Figure 6f). MiR-545-3p mimics-mediated impacts on the protein expression of PCNA and Bax were hindered after PDPK1 overexpression (Figure 6g and h). Further, miR-545-3p mimic repressed the production of glutamine and glutamate and downregulated GLS1 protein expression, but these effects were restored by the enforced PDPK1 expression (Figure 6i–k). Thus, these results explained that miR-545-3p could suppress H522 and A549 cell tumor properties by binding to PDPK1.

### 3.7 circ_0000376 silencing restrained tumor formation *in vivo*

**Figure 7:**
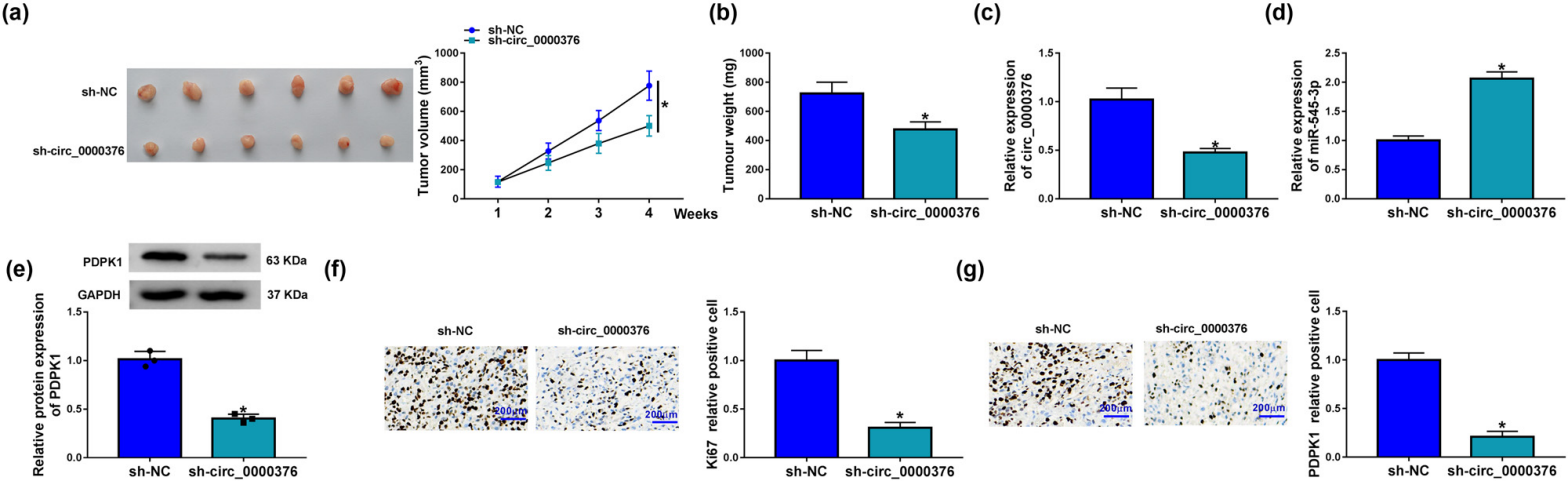
circ_0000376 knockdown inhibited tumor formation. (a and b) Impacts of circ_0000376 absence on tumor volume and weight were revealed. (c and d) Effects of circ_0000376 silencing on the expression of circ_0000376 and miR-545-3p were determined by qRT-PCR in tumor tissues of mice. (e) Influence of circ_0000376 downregulation on PDPK1 protein expression was analyzed by western blot in the tumor tissues of mice. (f and g) Protein expression of Ki67 and PDPK1 was determined by IHC assay. *P < 0.05.

To validate the effects of circ_0000376 on NSCLC progression *in vivo*, the study performed the xenograft mouse model assay. The results showed that circ_0000376 knockdown suppressed tumor volume and weight (Figure 7a and b), suggesting that circ_0000376 absence could inhibit tumor formation. Additionally, we found that circ_0000376 expression was decreased in the primary tumors from the sh-circ_0000376 group in comparison with those tumors from the sh-NC group (Figure 7c), implying that sh-circ_0000376 was effective in reducing circ_0000376 expression. Additionally, circ_0000376 knockdown upregulated miR-545-3p expression (Figure 7d) but downregulated PDPK1 protein expression (Figure 7e) in the primary tumors. The number of Ki67 or PDPK1 positive cells was fewer in the sh-circ_0000376 group than in the sh-NC group (Figure 7f and g), suggesting that circ_0000376 knockdown suppressed the protein expression of Ki67 and PDPK1 in the primary tumors. These data demonstrated that circ_0000376 absence inhibited tumor formation by regulating miR-545-3p and PDPK1.

## 4 Discussion

The special structure of circRNAs makes them resist ribonuclease, suggesting the potential of circRNAs as biological biomarkers for cancers [28]. As reported, NSCLC progression involves the dysregulation of circRNAs [29]. For instance, the increased expression of circ_0074027 contributed to NSCLC cell proliferation and metastasis by interacting with miR-185-3p [30]. Circ_0016760 overexpression promoted NSCLC cell metastatic property and inhibited cell apoptosis by binding to miR-1287 [31]. Circ_0001649 hindered NSCLC cell growth and metastasis by associating with miR-331-3p [32]. Based on the above evidence, we analyzed circRNA(s) related to the pathogenesis of NSCLC to provide a reliable therapeutic target for NSCLC. As a result, we found that circ_0000376 silencing repressed NSCLC cell tumor properties by the miR-545-3p/PDPK1 axis.

In this article, circ_0000376 was overexpressed in NSCLC specimens and cells. circ_0000376 was significantly associated with lymph node metastasis, TNM stage as well as tumor size of NSCLC patients. circ_0000376 absence inhibited NSCLC cell proliferation and metastasis. Additionally, circ_0000376 suppressed tumor formation *in vivo*. Previous data have suggested that circ_0000376 is augmented in NSCLC tissue samples and promotes NSCLC cell proliferation as well as metastasis [33]. Additionally, it was found that circ_0000376 knockdown reversed hypoxia-induced NSCLC cell proliferation and metastasis [15], implying that circ_0000376 could accelerate cell proliferation and metastasis. Our results were in line with the above data. Beyond that, our evidence also suggested that circ_0000376 absence repressed glutamate and glutamine levels and induced cell apoptosis in NSCLC cells. GLS1 is a cancer-specific glutaminase and catalyzes the glutamine conversion into α-ketoglutarate. Recent work found that GLS1 was overexpressed in most NSCLC cells [34] and that GLS1 depletion reduced NSCLC cell growth [35]. Moreover, GLS1 splice variant GAC was essential for NSCLC cell growth [36]. Herein, we found that circ_0000376 depletion reduced GLS1 protein expression. Considering that circRNA commonly modulated cancer progression by sponging miRNA [37], we further analyzed circ_0000376-associated miRNA. As a result, we confirmed that circ_0000376 bound to miR-545-3p in NSCLC cells.

As reported, miR-545-3p repressed osteogenesis by interacting with LDL receptor related protein 5 [38] and cannabinoid receptor 2 [39]. Besides, miR-545-3p inhibited angiogenesis and metastasis of endometrial carcinoma [40]. In lung cancer, miR-545-3p weakened cell proliferative and metastatic abilities and upregulated cell apoptotic rate [41]. Besides, circ_0072083 sponged miR-545-3p to regulate cisplatin-triggered promotion of NSCLC cell proliferation and metastasis and repression of cell apoptosis [42], suggesting that miR-545-3p inhibited NSCLC cell tumor properties. Similarly, our results showed that miR-545-3p was decreased in NSCLC clinical samples as well as cell lines and that miR-545-3p hindered NSCLC cell tumor properties through the regulation of cell proliferation, metastasis, and apoptosis. Different from the published data, we reported the repressing impact of miR-545-3p on glutamine metabolism for the first time. Additionally, we demonstrated that circ_0000376 regulated NSCLC tumorigenesis by interacting with miR-545-3p.

PDPK1 can activate many downstream factors related to the process of diseases, including cancer [43]. PDPK1 overexpression could neutralize solamargine-reduced NSCLC cell growth [44]. In addition, PDPK1 was associated with the inhibitory effect of Berberine on NSCLC cell proliferation [45]. In this article, PDPK1 was identified as a target of miR-545-3p. A dramatic high expression of PDPK1 was observed in NSCLC clinical samples and cell lines. Additionally, PDPK1 overexpression impaired miR-545-3p mimic-mediated NSCLC cell phenotypes, suggesting that PDPK1 promoted cell proliferation and metastasis and repressed cell apoptosis, which was further proved by Zhou et al. [25] and Li and his colleagues [46]. Beyond that, our data also showed that PDPK1 could promote glutamine catabolism and that miR-545-3p regulated NSCLC tumorigenesis through PDPK1. Further, circ_0000376 regulated PDPK1 expression by interacting with miR-545-3p. Meanwhile, *in vivo* assay showed that circ_0000376 absence downregulated PDPK1 expression in xenograft.

In addition, *in vivo* assay related to the effects of miR-545-3p depletion, circ_0000376 overexpression, or PDPK1 depletion on tumor growth can further validate the regulatory effects of the circ_0000376/miR-545-3p/PDPK1 axis on NSCLC cell phenotypes. This issue should be considered when evaluating the present study.

**Figure 8:**
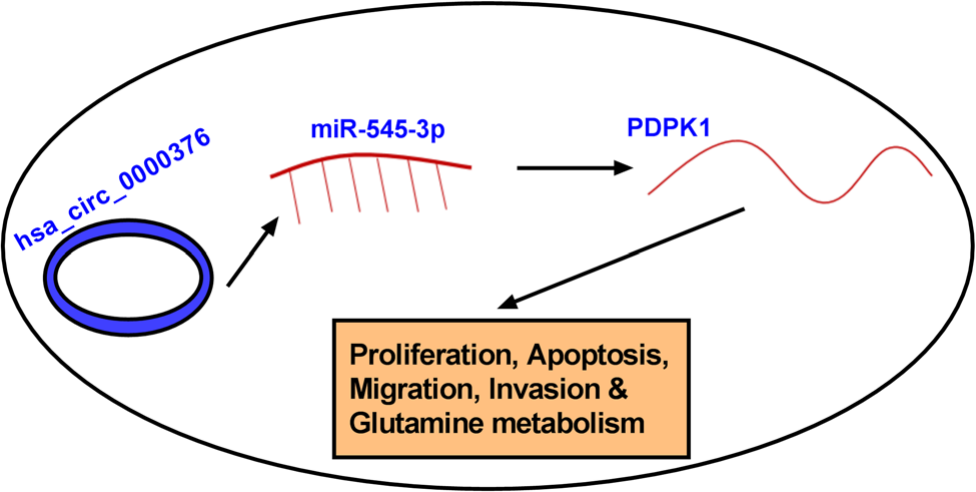
The schematic illustration showing the mechanism underlying circ_0000376 regulating NSCLC cell proliferation, migration, invasion, apoptosis, and glutamine metabolism.

All in all, the present study found that circ_0000376 expression was upregulated in NSCLC tissues and cells and its silencing repressed NSCLC cell tumor properties. Additionally, circ_0000376 absence repressed NSCLC process by binding to miR-545-3p *in vitro*. MiR-545-3p also regulated NSCLC cell development by targeting PDPK1. Collectively, circ_0000376 regulated NSCLC cell tumor properties by the miR-545-3p/PDPK1 pathway (Figure 8). Our results suggest the potential of circ_0000376 as a novel therapeutic target for NSCLC.

## Supplementary Material

Supplementary Figure

## References

[1] Torre LA, Bray F, Siegel RL, Ferlay J, Lortet-Tieulent J, Jemal A. Global cancer statistics, 2012. CA Cancer J Clin. 2015;65(2):87–108. 10.3322/caac.21262 25651787

[2] Gu Y, Pei X, Ren Y, Cai K, Guo K, Chen J, et al. Oncogenic function of TUSC3 in non-small cell lung cancer is associated with Hedgehog signalling pathway. Biochim Biophys Acta Mol Basis Dis. 2017;1863(7):1749–60. 10.1016/j.bbadis.2017.05.005 28487226

[3] Siegel RL, Miller KD, Jemal A. Cancer statistics, 2019. CA Cancer J Clin. 2019;69(1):7–34. 10.3322/caac.21551 30620402

[4] Antonia SJ, Balmanoukian A, Brahmer J, Ou SI, Hellmann MD, Kim SW, et al. Clinical activity, tolerability, and long-term follow-up of durvalumab in patients with advanced NSCLC. J Thorac Oncol. 2019;14(10):1794–806. 10.1016/j.jtho.2019.06.010 31228626

[5] Li L, Wan K, Xiong L, Liang S, Tou F, Guo S. CircRNA hsa_circ_0087862 acts as an oncogene in non-small cell lung cancer by targeting miR-1253/RAB3D axis. OncoTargets Ther. 2020;13:2873–86. 10.2147/OTT.S243533 PMC7138622 32308420

[6] Kristensen LS, Andersen MS, Stagsted LVW, Ebbesen KK, Hansen TB, Kjems J. The biogenesis, biology and characterization of circular RNAs. Nat Rev Genet. 2019;20(11):675–91. 10.1038/s41576-019-0158-7 31395983

[7] Hansen TB, Jensen TI, Clausen BH, Bramsen JB, Finsen B, Damgaard CK, et al. Natural RNA circles function as efficient microRNA sponges. Nature. 2013;495(7441):384–8. 10.1038/nature11993 23446346

[8] Verduci L, Strano S, Yarden Y, Blandino G. The circRNA-microRNA code: emerging implications for cancer diagnosis and treatment. Mol Oncol. 2019;13(4):669–80. 10.1002/1878-0261.12468 PMC6441890 30719845

[9] Lu J, Zhang P-Y, Xie J-W, Wang J-B, Lin J-X, Chen Q-Y, et al. Circular RNA hsa_circ_0006848 related to ribosomal protein L6 acts as a novel biomarker for early gastric cancer. Dis Markers. 2019;2019:3863458. 10.1155/2019/3863458 PMC6746163 31565098

[10] Shen C, Wu Z, Wang Y, Gao S, Da L, Xie L, et al. Downregulated hsa_circ_0077837 and hsa_circ_0004826, facilitate bladder cancer progression and predict poor prognosis for bladder cancer patients. Cancer Med. 2020;9(11):3885–903. 10.1002/cam4.3006 PMC7286451 32250047

[11] Wang G, Zhang H, Li P. Upregulation of hsa_circRNA_102958 indicates poor prognosis and promotes ovarian cancer progression through miR-1205/SH2D3A axis. Cancer Manag Res. 2020;12:4045–53. 10.2147/CMAR.S248560 PMC7264029 32547237

[12] Hong W, Xue M, Jiang J, Zhang Y, Gao X. Circular RNA circ-CPA4/let-7 miRNA/PD-L1 axis regulates cell growth, stemness, drug resistance and immune evasion in non-small cell lung cancer (NSCLC). J Exp Clin Cancer Res. 2020;39(1):149. 10.1186/s13046-020-01648-1 PMC7397626 32746878

[13] Ju C, Zhou J, Miao H, Chen X, Zhang Q. Bupivacaine suppresses the progression of gastric cancer through regulating circ_0000376/miR-145-5p axis. BMC Anesthesiol. 2020;20(1):275. 10.1186/s12871-020-01179-4 PMC7597012 33126850

[14] Peng Z, Xu B, Jin F. Circular RNA hsa_circ_0000376 participates in tumorigenesis of breast cancer by targeting miR-1285-3p. Technol Cancer Res Treat. 2020;19:1533033820928471. 10.1177/1533033820928471 PMC7257864 32462972

[15] Li C, Liu H, Niu Q, Gao J. Circ_0000376, a novel circRNA, promotes the progression of non-small cell lung cancer through regulating the miR-1182/NOVA2 network. Cancer Manag Res. 2020;12:7635–47. 10.2147/CMAR.S258340 PMC7455537 32922073

[16] Cui M, Yao X, Lin Y, Zhang D, Cui R, Zhang X. Interactive functions of microRNAs in the miR-23a-27a-24-2 cluster and the potential for targeted therapy in cancer. J Cell Physiol. 2020;235(1):6–16. 10.1002/jcp.28958 31192453

[17] Bica-Pop C, Cojocneanu-Petric R, Magdo L, Raduly L, Gulei D, Berindan-Neagoe I. Overview upon miR-21 in lung cancer: focus on NSCLC. Cell Mol Life Sci. 2018;75(19):3539–51. 10.1007/s00018-018-2877-x PMC11105782 30030592

[18] Petrek H, Yu AM. MicroRNAs in non-small cell lung cancer: gene regulation, impact on cancer cellular processes, and therapeutic potential. Pharmacol Res Perspect. 2019;7(6):e00528. 10.1002/prp2.528 PMC6923806 31859460

[19] Cai J, Fang L, Huang Y, Li R, Xu X, Hu Z, et al. Simultaneous overactivation of Wnt/β-catenin and TGFβ signalling by miR-128-3p confers chemoresistance-associated metastasis in NSCLC. Nat Commun. 2017;8:15870. 10.1038/ncomms15870 PMC5481840 28627514

[20] Liu X, Zhou X, Chen Y, Huang Y, He J, Luo H. miR-186-5p targeting SIX1 inhibits cisplatin resistance in non-small-cell lung cancer cells (NSCLCs). Neoplasma. 2020;67(1):147–57. 10.4149/neo_2019_190511N420 31686523

[21] He Z, Liu Y, Xiao B, Qian X. miR-25 modulates NSCLC cell radio-sensitivity through directly inhibiting BTG2 expression. Biochem Biophys Res Commun. 2015;457(3):235–41. 10.1016/j.bbrc.2014.12.094 25576360

[22] Chen S, Lu S, Yao Y, Chen J, Yang G, Tu L, et al. Downregulation of hsa_circ_0007580 inhibits non-small cell lung cancer tumorigenesis by reducing miR-545-3p sponging. Aging (Albany NY). 2020;12(14):14329–40. 10.18632/aging.103472 PMC7425484 32681720

[23] Zheng N, Ding X, Sun A, Jahan R. PDK1 activity regulates proliferation, invasion and growth of hemangiomas. Cell Physiol Biochem. 2015;36(5):1903–10. 10.1159/000430159 26202351

[24] Maegawa S, Chinen Y, Shimura Y, Tanba K, Takimoto T, Mizuno Y, et al. Phosphoinositide-dependent protein kinase 1 is a potential novel therapeutic target in mantle cell lymphoma. Exp Hematol. 2018;59:72–81e2. 10.1016/j.exphem.2017.12.006 29287939

[25] Zhou Y, Zheng X, Xu B, Chen L, Wang Q, Deng H, et al. Circular RNA hsa_circ_0004015 regulates the proliferation, invasion, and TKI drug resistance of non-small cell lung cancer by miR-1183/PDPK1 signaling pathway. Biochem Biophys Res Commun. 2019;508(2):527–35. 10.1016/j.bbrc.2018.11.157 30509491

[26] Cardano M, Tribioli C, Prosperi E. Targeting proliferating cell nuclear antigen (PCNA) as an effective strategy to inhibit tumor cell proliferation. Curr Cancer Drug Targets. 2020;20(4):240–52. 10.2174/1568009620666200115162814 31951183

[27] Liu Z, Ding Y, Ye N, Wild C, Chen H, Zhou J. Direct activation of bax protein for cancer therapy. Med Res Rev. 2016;36(2):313–41. 10.1002/med.21379 PMC4752390 26395559

[28] Chen Y, Li C, Tan C, Liu X. Circular RNAs: a new frontier in the study of human diseases. J Med Genet. 2016;53(6):359–65. 10.1136/jmedgenet-2016-103758 26945092

[29] Szabo L, Salzman J. Detecting circular RNAs: bioinformatic and experimental challenges. Nature Reviews Genetics. 2016;17(11):679–92. 10.1038/nrg.2016.114 PMC5565156 27739534

[30] Gao P, Wang Z, Hu Z, Jiao X, Yao Y. Circular RNA circ_0074027 indicates a poor prognosis for NSCLC patients and modulates cell proliferation, apoptosis, and invasion via miR-185-3p mediated BRD4/MADD activation. J Cell Biochem. 2020;121(3):2632–42. 10.1002/jcb.29484 31680319

[31] Li Y, Hu J, Li L, Cai S, Zhang H, Zhu X, et al. Upregulated circular RNA circ_0016760 indicates unfavorable prognosis in NSCLC and promotes cell progression through miR-1287/GAGE1 axis. Biochem Biophys Res Commun. 2018;503(3):2089–94. 10.1016/j.bbrc.2018.07.164 30103946

[32] Liu T, Song Z, Gai Y. Circular RNA circ_0001649 acts as a prognostic biomarker and inhibits NSCLC progression via sponging miR-331-3p and miR-338-5p. Biochem Biophys Res Commun. 2018;503(3):1503–9. 10.1016/j.bbrc.2018.07.070 30029881

[33] Sun H, Chen Y, Fang YY, Cui TY, Qiao X, Jiang CY, et al. Circ_0000376 enhances the proliferation, metastasis, and chemoresistance of NSCLC cells via repressing miR-384. Cancer Biomark. 2020;29(4):463–73. 10.3233/CBM-190912 PMC12662546 32716343

[34] Lee J-S, Kang JH, Lee S-H, Hong D, Son J, Hong KM, et al. Dual targeting of glutaminase 1 and thymidylate synthase elicits death synergistically in NSCLC. Cell Death Dis. 2016;7(12):e2511. 10.1038/cddis.2016.404 PMC5261012 27929535

[35] Lee JS, Kang JH, Lee SH, Lee CH, Son J, Kim SY. Glutaminase 1 inhibition reduces thymidine synthesis in NSCLC. Biochem Biophys Res Commun. 2016;477(3):374–82. 10.1016/j.bbrc.2016.06.095 27338638

[36] van den Heuvel AP, Jing J, Wooster RF, Bachman KE. Analysis of glutamine dependency in non-small cell lung cancer: GLS1 splice variant GAC is essential for cancer cell growth. Cancer Biol Ther. 2012;13(12):1185–94. 10.4161/cbt.21348 PMC3469476 22892846

[37] Tang Q, Hann SS. Biological roles and mechanisms of circular RNA in human cancers. OncoTargets Ther. 2020;13:2067–92. 10.2147/OTT.S233672 PMC7069569 32210574

[38] Li L, Qiu X, Sun Y, Zhang N, Wang L. SP1-stimulated miR-545-3p inhibits osteogenesis via targeting LRP5-activated Wnt/beta-catenin signaling. Biochem Biophys Res Commun. 2019;517(1):103–10. 10.1016/j.bbrc.2019.07.025 31327495

[39] Hao R, Wang B, Wang H, Huo Y, Lu Y. lncRNA TUG1 promotes proliferation and differentiation of osteoblasts by regulating the miR-545-3p/CNR2 axis. Braz J Med Biol Res. 2020;53(11):e9798. 10.1590/1414-431X20209798 PMC7552904 33053117

[40] Zhong Y, Wang Y, Dang H, Wu X. LncRNA AFAP1-AS1 contributes to the progression of endometrial carcinoma by regulating miR-545-3p/VEGFA pathway. Mol Cell Probes. 2020;53:101606. 10.1016/j.mcp.2020.101606 32504788

[41] Sun J, Min H, Yu L, Yu G, Shi Y, Sun J. The knockdown of LncRNA AFAP1-AS1 suppressed cell proliferation, migration, and invasion, and promoted apoptosis by regulating miR-545-3p/hepatoma-derived growth factor axis in lung cancer. Anticancer Drugs. 2020;32(1):11–21. 10.1097/CAD.0000000000001003 33290312

[42] Li H, Liu F, Qin W. Circ_0072083 interference enhances growth-inhibiting effects of cisplatin in non-small-cell lung cancer cells via miR-545-3p/CBLL1 axis. Cancer Cell Int. 2020;20:78. 10.1186/s12935-020-1162-x PMC7066755 32190002

[43] Gagliardi PA, di Blasio L, Primo L. PDK1: a signaling hub for cell migration and tumor invasion. Biochim Biophys Acta. 2015;1856(2):178–88. 10.1016/j.bbcan.2015.07.003 26238471

[44] Tang Q, Zheng F, Liu Z, Wu J, Chai X, He C, et al. Novel reciprocal interaction of lncRNA HOTAIR and miR-214-3p contribute to the solamargine-inhibited PDPK1 gene expression in human lung cancer. J Cell Mol Med. 2019;23(11):7749–61. 10.1111/jcmm.14649 PMC6815775 31475459

[45] Zheng F, Wu J, Tang Q, Xiao Q, Wu W, Hann SS. The enhancement of combination of berberine and metformin in inhibition of DNMT1 gene expression through interplay of SP1 and PDPK1. J Cell Mol Med. 2018;22(1):600–12. 10.1111/jcmm.13347 PMC5742731 28840963

[46] Li C, Zhao W, Pan X, Li X, Yan F, Liu S, et al. LncRNA KTN1-AS1 promotes the progression of non-small cell lung cancer via sponging of miR-130a-5p and activation of PDPK1. Oncogene. 2020;39(39):6157–71. 10.1038/s41388-020-01427-4 32820252

