# Supplementary material for: circ_0000376 knockdown suppresses non-small cell lung cancer cell tumor properties by the miR-545-3p/PDPK1 pathway
Source: Open Med (Wars). 2023 Feb 16;18(1):20230641. doi: 10.1515/med-2023-0641 (PMC9938644; doi:10.1515/med-2023-0641)
Supplement: Supplementary Figure [file med-2023-0641-sm.pdf]

Supplementary material

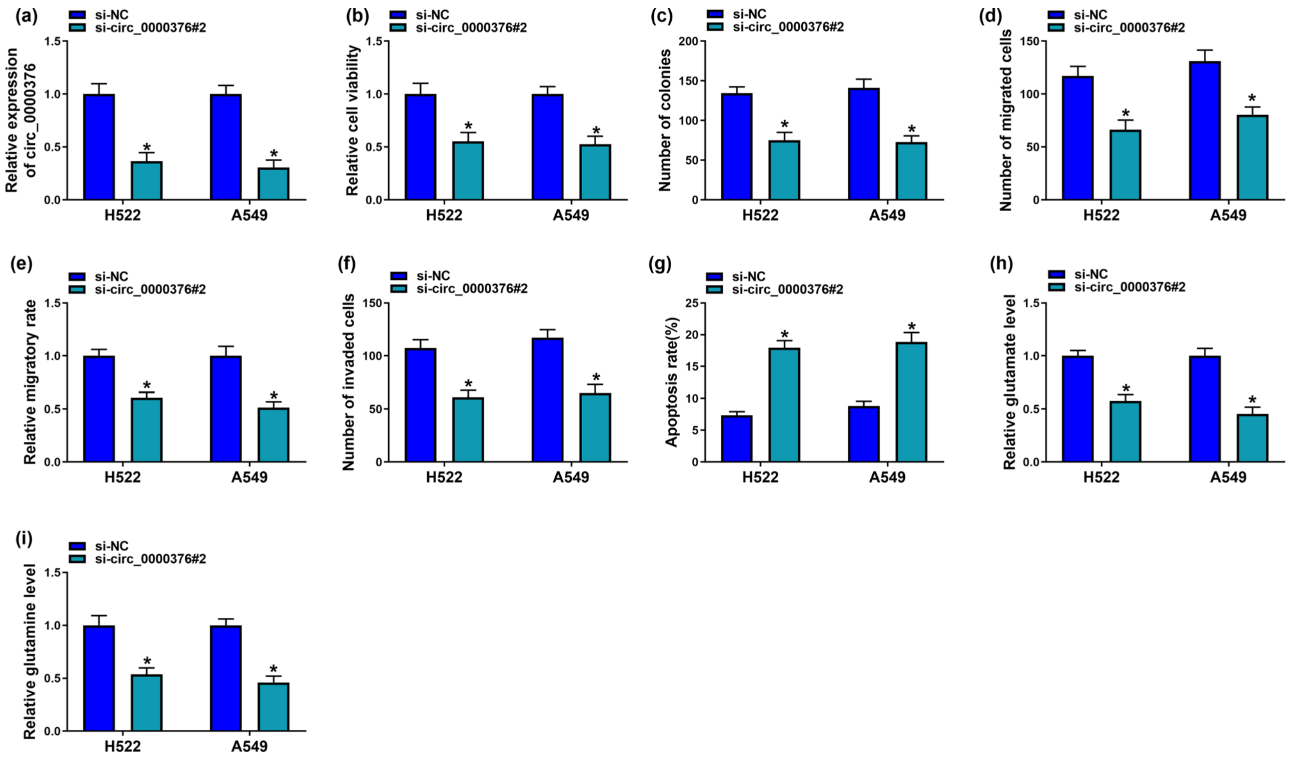

Figure S1: Circ_0000376 absence repressed NSCLC cell tumor properties. a–i) Both H522 and A549 cells were transfected with si-NC and si-circ_0000376#2, respectively. (a) The interfering efficiency of si-circ_0000376#2 was determined by qRT-PCR. (b and c) Cell viability and colony-forming ability were detected by CCK-8 and colony formation assays, respectively. (d and e) Transwell migration and wound-healing assays were performed to analyze the migratory ability of H522 and A549 cells. (f) Transwell invasion assay was carried out to determine the invasive ability of H522 and A549 cells. (g) Cell apoptosis was detected by flow cytometry analysis. (h and i) The levels of glutamate and glutamine were determined by glutamate detection and glutamine determination assays, respectively. *P<0.05.
